# Case Report: Hematoma Formation After Spontaneous Coronary Artery Rupture

**DOI:** 10.3389/fcvm.2021.801005

**Published:** 2022-01-11

**Authors:** Weitao Liang, Honghua Yue, Tailong Zhang, Zhong Wu

**Affiliations:** ^1^Department of Cardiovascular Surgery, West China Hospital, Sichuan University, Chengdu, China; ^2^Union Hospital Affiliated With Huazhong University of Science and Technology, Wuhan, China

**Keywords:** spontaneous coronary artery rupture, coronary artery hematoma, cardiac mass, coronary aneurysm, literature review

## Abstract

We report a case of hematoma formation in the right coronary artery after spontaneous rupture. A 48-year-old female patient was admitted with a suspected right cardiac mass. Despite diagnostic work-up, the dignity of the mass could not be determined. Due to acute clinical symptoms, explorative surgery was decided and performed. Hereby, the mass was partially incised, and thrombus-like tissue was detected without active bleeding. We described the challenges during the diagnostic process, and the diagnosis was finally made according to a multimodality approach. For further assessment, we reviewed related literature and highlighted the importance of coronary angiography in the preoperative evaluation of such patients. The therapy may vary according to the location and size of such lesions.

## Introduction

Coronary artery rupture is the rupture of the coronary vessels, which is usually secondary to coronary aneurysm, dissection, trauma, connective tissue diseases (Marfan syndrome), vasculitis (Kawasaki disease, Takayasu's arteritis), heart tumor, infective endocarditis, or iatrogenic intervention. Some reports described scarce etiologies, such as amphetamine abuse (1), intraoperative fracture (2), or highly active antiretroviral therapy (3). However, spontaneous coronary artery rupture (SCAR) is a rare but severe entity. Without a proper diagnosis and treatment, it may lead to unfavorable results relating to cardiac tamponade, heart failure, and sudden death. We present a case of SCAR due to hematoma formation and a review of the related literature, summarizing the current clinical experience in this field.

## Case Report

**Figure 1:**
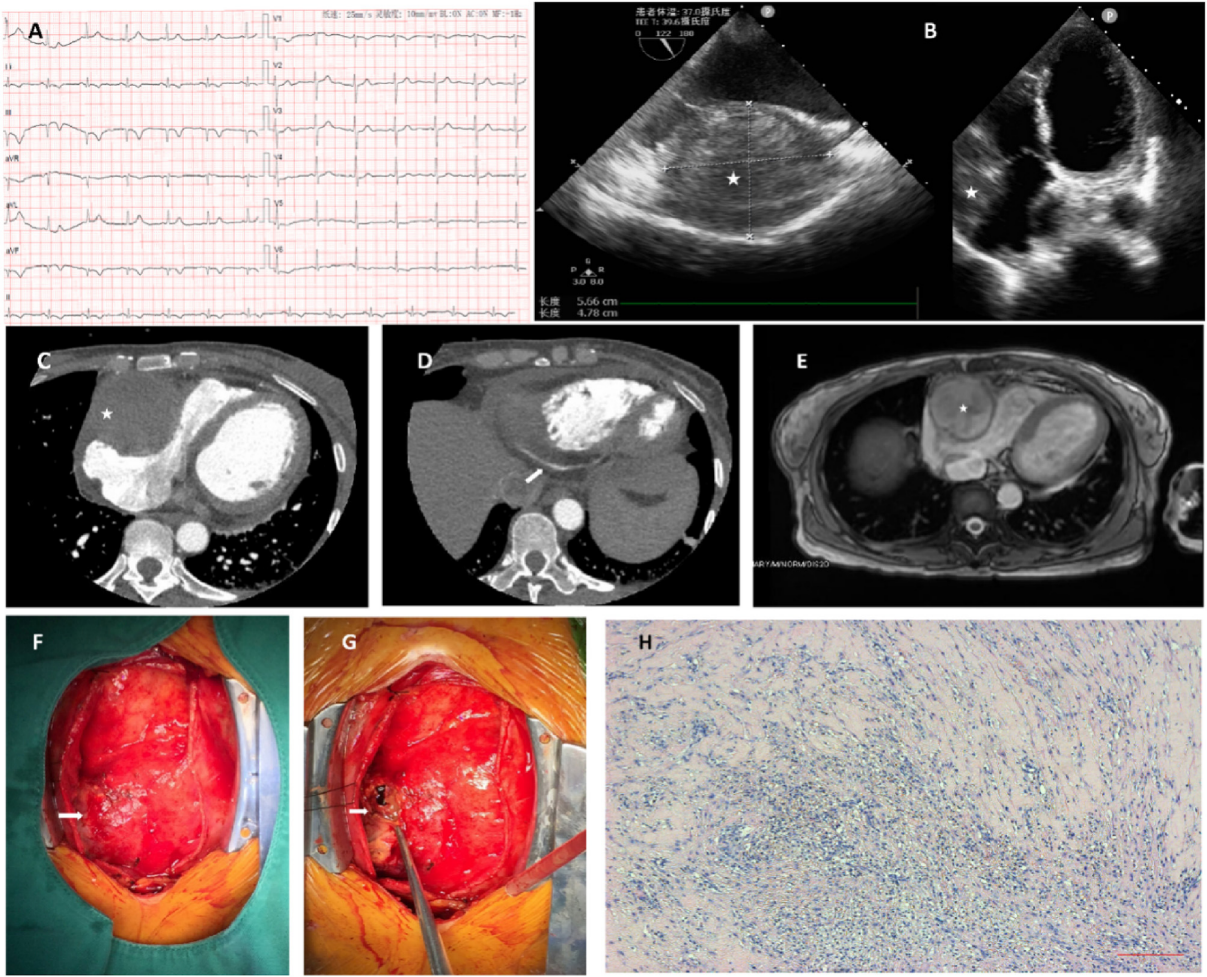
**(A)** ECG shows Q-waves in II, III, aVF leads, and left axis deviation, indicating inferior myocardial infarction. **(B)** A large (59 × 53 mm), heterogeneous mass detected on transthoracic echocardiography (TTE) located in the right atrioventricular groove. **(C)** Computed tomographic angiography (CTA) shows a spherical, mixed-density mass (5.4 × 5.5 cm) located at the anterior wall of the right atrium. **(D)** With coronary artery CTA, the proximal right coronary artery is not possible. Contrast filling can be detected in the distal part of the artery. **(E)** Cardiac MRI manifests a large (5.6 × 5.1 × 5. cm), round, mixed-signal mass located in the right atrioventricular groove. The right atrium and the right ventricle are compressed, and the ejection fractions (EFs) of the left and right ventricles are 36.1 and 40.1%, respectively. **(F)** The oval, cystic, under epicardium hematoma, is revealed out-punching from the right atrioventricular groove through pericardiotomy. The hematoma was partially incised, and the thrombus and abscess were found and evacuated. **(G)** The histopathological report demonstrates fibrosis with the additional presence of inflammatory cells. **(H)** The content was thrombus, with a plethora of neutrophil granulocytes inside.

A 48-year-old woman presented with recurrent, mild, dull chest pain over 4 months and had a sudden right-sided chest pain attack with dyspnea and sweating 2 weeks prior. The patient had no significant previous medical history or family history. No murmur or other abnormalities were found during physical examination. Sinus rhythm was present with ECG, which also showed Q-waves in II, III, and aVF and a left axis deviation (Figure 1A). Her brain natriuretic peptide (BNP), troponin (TnT), and D-dimer levels were elevated (1,079 pg/ml, 71.8 62 ng/L and 4.13 mg/l FEU, respectively), while other laboratory noinoi were unremarkable. Transthoracic echocardiography (TTE) revealed a large (59 × 53 mm) heterogeneous mass in the right atrioventricular groove (Figure 1B). Computed tomographic angiography (CTA) showed a spherical, mixed-density mass (5.4 × 5.5 cm) located at the anterior wall of the right atrium (Figure 1C). For further assessment, coronary artery CTA was performed. Therefore, the proximal right coronary artery (RCA) could not be clearly visualized due to the presence of the tumor, whereas the distal right coronary artery and all other coronary arteries were patent without the presence of lumen narrowing or significant plaques (Figure 1D). Cardiac magnetic resonance imaging (MRI) manifested a large (5.6 × 5.1 × 5 cm), round, mixed-signal mass located in the right atrioventricular groove, compressed right heart, and ejection fractions (EFs) of the left and right ventricles of 36.1 and 40.1%, respectively (Figure 1E).

As the mass caused compression of the right heart and the risk of rupture, we decided to proceed with a median sternotomy. Neither hemopericardium nor pericardial effusion was found during the operation. The lesion was located at the right atrioventricular groove. After resection of the outer layer of the lesion, thrombus-like tissue was found, which was consistent with the diagnosis of hematoma formation caused by rupture of the coronary artery (Figures 1F,G). It should be noted that no atherosclerotic plaque was found during the operation, indicating that it probably formed spontaneously and not due to plaque rupture. Since there was no active bleeding, we did not ligate the nearby artery, and the whole procedure was performed on a beating heart. The lumen was then cleaned with saline, evacuated, and closed with sutures. The histopathological study afterward indicated thrombus with accumulation of neutrophil granulocytes, while fibrous tissue was found in the mass wall with inflammatory cell infiltration. The patient had an uneventful recovery. Coronary CT and TTE showed relief of right heart compression, and the distal RCA was unobstructed. She was discharged from the hospital 5 days after surgery and remained well during the half-year follow-up after the treatment.

## Discussion

The diagnosis and treatment of cardiac masses can be challenging. Lesions may derive from neoplasms, aneurysms, vasculitis, or hematomas. Patients may present with unspecific symptoms ranging from asymptomatic angina, exercise intolerance to respiratory syndromes, or even sudden death according to the location and characteristics of the lesion. In this case, the diagnosis may be cumbersome. The patient presented with angina, and the ECG showed possible myocardial infarction. Coronary artery atherosclerosis was considered, while thoracic CT revealed a large mass located at the right heart. CTA demonstrated none enhancement of the entire mass, which ruled out the possibility of aneurysm or pseudoaneurysm. The distal RCA was unobstructed, and its proximal segment remained unclear simultaneously. Based on this point and in addition, no clinical features or medical history that suggested connective tissue disease or inflammation, which was related to vasculitis, a neoplasm of the right atrium was considered. Cardiac MRI proved the existence of myocardial infarction of the left ventricular inferior wall; compression led to a decreased EF of the right ventricle, as we presumed. However, it was possible to be correlated with potential myocarditis or ischemic injury.At this stage, the absence of cardiac tamponade symptoms and the relatively stable state of the patient made it even harder to recognize the existence of coronary artery rupture; thus, we decidedto perform an exploratory surgery to determine the properties of the mass. During the operation, the mass was partially incised, and a large amount of organized thrombi and histopathological findings was consistent with the diagnosis of coronary artery rupture. However, there was still diagnostic uncertainty as to the nature of the mass, since no active bleeding of the coronary artery was found during the operation. As long as we considered, thrombosis and organization spontaneously restricted further bleeding of the right coronary artery after rupture. The cause of the rupture remained unknown. The hypothesis of the former existence of a coronary aneurysm or false aneurysm that ruptured could not be excluded. Generally, coronary artery rupture may be caused by catheter-based intervention, vasculitis, infections and trauma, while spontaneous rupture with the absence of all these medical histories, similar to this case, is rare. The natural history of SCAR is poorly defined, which increases the difficulty of clinical diagnosis (4). For further assessment, we reviewed related cases reported as spontaneous coronary artery rupture listed in Table 1.

**Table 1:** Related cases reported as spontaneous coronary artery rupture in the literature.

**References**	**Gender and age**	**Symptoms**	**Lesion location**	**Bleeding**	**Tamponade**	**Intervention**	**Prognosis**
Kaljusto et al. (5)	M, 50	Fatigue, pain, fever and hypotension	RCA	Yes	Yes	Evacuation, CPB, ligation	Favorable
Kaljusto et al. (5)	M, 62	Pain, tachycardia, hypotension	Diagonal	No	Yes	Pericardial patch	Favorable
Kaljusto et al. (5)	M, 69	Pain, hypotension	Diagonal	Yes	Yes	Pericardiocentesis, stent, CPB, pericardial patch, CABG	Favorable
Butz et al. (6)	M, 65	Acute heart failure	RCA	No	Yes	Evacuation, CABG	NA
He et al. (7)	M, 58	Hypotension, tachycardia, dyspnea, cyanosis	LCX	Yes	Yes	Pericardiocentesis, ligation	Dead
Longobardi et al. (8)	M, 37	Fatigue, pain, hypotension	RCA	Yes	Yes	CPB, ligation, surture	Favorable
Kim (9)	M, 67	Cardiac arrest, shock	Ramus intermedius	Yes	Yes	Suture repair	Favorable
Sevuk et al. (10)	M, 60	Pain, nausea, vomiting	RCA	No	No	CPB, ligation, CABG	Favorable
Moizumi et al. (11)	M, 69	Epigalstragia, shock	LCX	Yes	Yes	Pericardiocentesis, CPB, ligation, CABG	Favorable
Hansch et al. (12)	F, 65	Dyspnea, heart failure	RCA	Yes	No	Ligation, CABG	NA
Kim et al. (13)	M, 28	Pain, breath shortness	RCA	No	No	Hematoma incision, suture	Favorable
Moonen et al. (14)	M, 56	Hypotension, tachycardia, dyspnea, cyanosis	Posterior interventricular artery	Yes	Yes	Ligation	Favorable

In reported cases, SCAR occurred mostly in over 50-year-old males with clinical symptoms ranging from fatigue, chest pain, tachycardia, dyspnea to hypotension, and heart failure. In most cases, continuous coronary bleeding exists, which always results in tamponade except for one case (12), where tamponade did not occur. In some cases (5, 6, 10, 13), there was no continuous coronary artery bleeding in which two of them (5, 6), and in two of them, tamponade remained. Two cases (10, 13) described patients with SCAR without effusion, tamponade, or bleeding. The culprit vessels were different, while spontaneous main LCA or left anterior descending artery (LAD) rupture remained unreported. The treatment methods vary depending on specific factors. Due to the risk of bleeding and tamponade, some cases require emergency treatment, including pericardiocentesis and surgical intervention. Sternotomy was performed in all cases reported; the surgical intervention included cardiopulmonary bypass (CPB), hematoma incision, evacuation, pericardial patch, ruptured artery suture or ligation, and CABG. Most of the patients in these cases reported a favorable prognosis except for one death (7) due to low cardiac output (no myocardial ischemia was found) and acute renal failure after surgery.

Due to a low incidence rate, the correct diagnosis of SCAR is challenging. Differential diagnosis includes CAD, thoracic artery dissection, aneurysm, and cardiac tumors. Thoroughly, history taking is necessary. Meanwhile, thoracic CT scans and TTE are fast and convenient examinations that may reveal the existence of hematoma or hemopericardium. Coronary angiography plays a crucial role in revealing the artery condition directly. However, patients presenting with unstable hemodynamic diagnostic work-ups need to be time efficient. Thus, in some such cases, cardiac CT or even coronary angiography may not be possible in the interest of time. MRI is precious to evaluate the properties of cardiac mass, myocardial perfusion, and heart movement, which should only be used in patients with stable vital signs. In addition, cardiac CT is a quick and practical method for the diagnostic work-up of such cases.

Surgical treatment varies according to the symptoms and hemodynamic state. For patients presenting with symptoms of cardiac tamponade (such as arterial pressure decreasing, venous pressure increasing, and blood pressure not being maintained), emergency surgery should be considered immediately to achieve tamponade drainage and hemostasis by evacuation of the hemopericardium, ligation of the culprit's vessel, or rupture repair. Nevertheless, for patients with a stable condition, as we described, a thorough examination should be performed preoperatively to achieve an accurate diagnosis. For the operation, rupture repair, hematoma incision, and CABG should be considered. CPB is not always needed in this situation if hemostasis and repair can be safely achieved after evaluation. We did not perform CABG, as coronary CTA showed contrast filling in the distal RCA prior to surgery. The patient remained coronary symptom free during the half-year follow-up, demonstrating that CABG surgery might not always be necessary for SCAR. However, reviewing our diagnosis process of this case, the strategy could have been more precise if coronary angiography was performed in the first place, which would reveal the relationship between the hematoma and coronary artery.

## Conclusion

Coronary artery rupture may occur in many situations. Although SCAR is rare, the diagnosis deserves a high level of suspicion. Hematoma formation after SCAR may confuse the diagnosis with cardiac mass. Therefore, we recommend coronary angiography for any conditional patient, which plays a crucial part in the diagnosis and treatment strategy for SCAR. The treatment varies as long as the aim of hemostasis and cardiac function protection can be achieved. According to the lesion and bleeding conditions, the operational intervention includes hematoma resection, evacuation, culprit vessel ligation, suture repair, and myocardial revascularization. Cardiopulmonary bypass is not always necessary.

## Data Availability Statement

The raw data supporting the conclusions of this article will be made available by the authors, without undue reservation.

## Ethics Statement

The authors are accountable for all aspects of the work in ensuring that questions related to the accuracy or integrity of any part of the work are appropriately investigated and resolved. All procedures performed in studies involving human participants were in accordance with the ethical standards of the Institutional Research Committee and with the Helsinki Declaration (as revised in 2013). Written informed consent was obtained from the patient.

## Author Contributions

WL and HY: conceptualization, investigation, and writing—original draft. TZ: methodology. ZW: conceptualization and writing—review and editing. All authors contributed to the article and approved the submitted version.

## Funding

This study was funded by the National Natural Science Foundation of China (Grant No. 82172060) and the 1.3.5 Project for Disciplines of Excellence-Clinical Research Incubation Project, West China Hospital, Sichuan University, 2020HXFH042.

## Conflict of Interest

The authors declare that the research was conducted in the absence of any commercial or financial relationships that could be construed as a potential conflict of interest.

## Publisher's Note

All claims expressed in this article are solely those of the authors and do not necessarily represent those of their affiliated organizations, or those of the publisher, the editors and the reviewers. Any product that may be evaluated in this article, or claim that may be made by its manufacturer, is not guaranteed or endorsed by the publisher.

## References

[1] Brennan K Shurmur S Elhendy A. Coronary artery rupture associated with amphetamine abuse. Cardiol Rev. (2004) 12:282–3. 10.1097/01.crd.0000132372.38506.45 15316310

[2] Borger MA David TE Doll N Mohr FW. Intraoperative fracture of the right coronary artery: recognition and management. Ann Thorac Surg. (2005) 79:693–6. 10.1016/j.athoracsur.2003.09.121 15680866

[3] De Giorgio F Abbate A Capelli A Arena V. Spontaneous rupture of coronary artery in human immunodeficiency virus-positive patient treated with highly active anti-retroviral therapy (HAART). Am J Forensic Med Pathol. (2005) 26:197. 10.1097/01.paf.0000163830.10327.60 15894860

[4] Kar S Webel RR. Diagnosis and treatment of spontaneous coronary artery pseudoaneurysm: rare anomaly with potentially significant clinical implications. Catheter Cardiovasc Interv. (2017) 90:589–97. 10.1002/ccd.26997 28258964

[5] Kaljusto ML Koldsland S Vengen OA Worldbaek PR Tønnessen T. Cardiac tamponade caused by acute spontaneous coronary artery rupture. J Card Surg. (2006) 21:301–3. 10.1111/j.1540-8191.2006.00239.x 16684069

[6] Butz T Lamp B Figura T Faber L Esdorn H Wiemer M. Images in cardiovascular medicine. Pericardial effusion with beginning cardiac tamponade caused by a spontaneous coronary artery rupture. Circulation. (2007) 116:e383–4. 10.1161/CIRCULATIONAHA.107.710913 17938293

[7] He Z Chen G He X He X. Spontaneous coronary artery rupture causing acute cardiac tamponade and cardiogenic shock. Int Heart J. (2019) 60:1009–12. 10.1536/ihj.18-432 31204372

[8] Longobardi A Iesu S Baldi C Di Maio M Panza A Mastrogiovanni G. Spontaneous coronary artery rupture presenting as an acute coronary syndrome evolved in pseudoaneurysm and cardiac tamponade: case report and literature review. Eur Heart J Acute Cardiovasc Care. (2017) 6:666–9. 10.1177/2048872615617043 26566773

[9] Kim KH. Spontaneous coronary artery rupture treated on a beating heart. J Card Surg. (2019) 34:1656–8. 10.1111/jocs.14262 31563138

[10] Sevuk U Ozyalcin S Ayaz F Kose K. Spontaneous coronary artery rupture without a pericardial effusion: a diagnostic challenge. BMJ Case Rep. (2016) 2016:214424. 10.1136/bcr-2016-214424 27055462 PMC4840701

[11] Moizumi Y Komatsu T Motoyoshi N Tabayashi K. Management of patients with intramural hematoma involving the ascending aorta. J Thorac Cardiovasc Surg. (2002) 124:918–24. 10.1067/mtc.2002.125637 12407374

[12] Hansch A Betge S Pfeil A Mayer TE Wolf G Berhm B. Images in cardiovascular medicine. Spontaneous rupture of the right coronary artery. Circulation. (2010) 121:2692–3. 10.1161/CIRCULATIONAHA.109.924290 20566967

[13] Kim KH Choi JB Kim KS. Subepicardial hematoma compressing the right atrium: spontaneous rupture of the right coronary artery. Ann Thorac Surg. (2008) 86:e9. 10.1016/j.athoracsur.2008.09.062 19021964

[14] Moonen ML Hanssen M Radermecker MA Lancellotti P. The blue man: an unusual happy end of a spontaneous rupture of a coronary artery. Eur J Cardiothorac Surg. (2008) 34:1265–7. 10.1016/j.ejcts.2008.08.031 18848457

